# Evaluating Nanoparticulate Vaccine Formulations for Effective Antigen Presentation and T-Cell Proliferation Using an In Vitro Overlay Assay

**DOI:** 10.3390/vaccines12091049

**Published:** 2024-09-13

**Authors:** Dedeepya Pasupuleti, Priyal Bagwe, Amarae Ferguson, Mohammad N. Uddin, Martin J. D’Souza, Susu M. Zughaier

**Affiliations:** 1 Vaccine Nanotechnology Laboratory, Center for Drug Delivery Research, College of Pharmacy, Mercer University, Atlanta, GA 30341, USA; dedeepya.pasupuleti@live.mercer.edu (D.P.); amarae.ferguson@live.mercer.edu (A.F.); priyal.bagwe@live.mercer.edu (P.B.); uddin_mn@mercer.edu (M.N.U.); 2 College of Medicine, QU Health, Qatar University, Doha P.O. Box 2731, Qatar

**Keywords:** T-cell proliferation, overlay assay, cell-to-cell contact, antigen-presenting cell, dendritic cell, antigen presentation, in vitro vaccine screening, CFSE

## Abstract

Inducing T lymphocyte (T-cell) activation and proliferation with specificity against a pathogen is crucial in vaccine formulation. Assessing vaccine candidates’ ability to induce T-cell proliferation helps optimize formulation for its safety, immunogenicity, and efficacy. Our in-house vaccine candidates use microparticles (MPs) and nanoparticles (NPs) to enhance antigen stability and target delivery to antigen-presenting cells (APCs), providing improved immunogenicity. Typically, vaccine formulations are screened for safety and immunostimulatory effects using in vitro methods, but extensive animal testing is often required to assess immunogenic responses. We identified the need for a rapid, intermediate screening process to select promising candidates before advancing to expensive and time-consuming in vivo evaluations. In this study, an in vitro overlay assay system was demonstrated as an effective high-throughput preclinical testing method to evaluate the immunogenic properties of early-stage vaccine formulations. The overlay assay’s effectiveness in testing particulate vaccine candidates for immunogenic responses has been evaluated by optimizing the carboxyfluorescein succinimidyl ester (CFSE) T-cell proliferation assay. DCs were overlaid with T-cells, allowing vaccine-stimulated DCs to present antigens to CFSE-stained T-cells. T-cell proliferation was quantified using flow cytometry on days 0, 1, 2, 4, and 6 upon successful antigen presentation. The assay was tested with nanoparticulate vaccine formulations targeting *Neisseria gonorrhoeae* (CDC F62, FA19, FA1090), measles, H1N1 flu prototype, canine coronavirus, and Zika, with adjuvants including Alhydrogel^®^ (Alum) and AddaVax™. The assay revealed robust T-cell proliferation in the vaccine treatment groups, with variations between bacterial and viral vaccine candidates. A dose-dependent study indicated immune stimulation varied with antigen dose. These findings highlight the assay’s potential to differentiate and quantify effective antigen presentation, providing valuable insights for developing and optimizing vaccine formulations.

## 1. Introduction

T-cell-mediated adaptive immune responses, in conjunction with innate immune responses, contribute to the intricate complexity of the immune system. During the initiation of the immune response, briefly stimulated CD8+ T-cells undergo an atypical rapid succession of extensive cell divisions. Subsequently, they transition into a state of quiescence, giving rise to long-lasting memory cells that establish immunological memory, which retains the capability to re-engage in proliferation upon encountering subsequent immunological stimuli [1,2,3]. A vaccine formulation is considered successful when it elicits a higher safety profile and induces both immediate innate and subsequent protective adaptive immune responses [4]. Adaptive immune responses, including humoral and cellular responses, heavily depend on T-cell activation and proliferation. A vaccine’s efficiency is determined by its ability to induce successful antigen presentation by the antigen-presenting cells (APCs), such as macrophages and DC, to the T-cells, leading to their activation and proliferation against the specific antigen [4]. For the establishment of immunity, CD8+ T-cells, when transiently stimulated, engage in rapid and extensive rounds of cell division, forming quiescent, durable memory CD4+ T-cells [5]. These memory cells remain ready to proliferate upon encountering future immunological threats [3].

Vaccine nanoformulations represent a significant advancement in immunization strategies, leveraging nanoparticles to enhance vaccine delivery, stability, and efficacy [6]. These nanoformulations, including liposomes, polymeric nanoparticles, lipid nanoparticles, and virus-like particles, offer several advantages [7,8,9]. These formulations enhance uptake by APCs, protect antigens from environmental degradation, increase shelf life, and reduce cold chain requirements [10]. Additionally, they provide controlled release, ensuring prolonged immune stimulation and potentially reducing the need for booster doses [11]. Targeted delivery is another key benefit, as nanoparticles can be engineered to direct antigens to specific immune cells, such as dendritic cells, thereby enhancing antigen presentation and eliciting more robust immune responses [12]. Nanoformulations also enable the incorporation of multiple antigens, providing broader protection against various strains or pathogens [13]. Furthermore, by controlling the release and localization of antigens, these formulations can minimize systemic side effects, improving safety profiles.

In developing nanoformulated vaccines, preclinical testing is a critical phase that involves evaluating safety, immunogenicity, and efficacy in animal models before progressing to human trials [14]. This phase includes in vitro assays to assess antigen stability, cellular uptake, and immunogenicity and in vivo studies to evaluate the safety, toxicity, pharmacokinetics, biodistribution, and immune responses to ensure both safety and effectiveness [15]. Moreover, improperly tested vaccines might induce severe side effects, such as induction of auto-antibodies, autoimmunity, or neurological disorders such as Guillain–Barré syndrome [16,17,18,19]. Additionally, the adjuvant activity of the nanoformulations is assessed to determine their ability to enhance immune responses compared to traditional adjuvants.

In vitro models that mimic the immune system are increasingly utilized as platforms to assess vaccine formulations [20,21,22]. The overlay assay, facilitating direct cell-to-cell contact between innate immune cells such as macrophages/dendritic cells and adaptive immune cells such as T-cells, mimics natural immune environments crucial for studying vaccine immunogenicity, stability, and consistency. These models evaluate cellular antigen uptake, assess antigen presentation efficiency, and quantitatively measure adaptive immune responses. Developing these accelerated high-throughput assays enhances vaccine formulation refinement, reduces reliance on animal testing, and accelerates vaccine development timelines.

In our efforts to develop an overlay assay for quantifying T-cell responses against vaccine candidates, the CFSE assay was optimized. CFSE, initially a non-fluorescent pro-dye, becomes a highly fluorescent carboxyfluorescein succinimidyl ester within cells through intracellular esterase activity, detectable via flow cytometry. CFSE fluorescence dilutes evenly among daughter cells as they divide, allowing quantitative assessment of immune cell proliferation in response to the vaccine challenge. In this assay, T-cells from the adaptive immune system are labeled with CFSE, stimulated with activated antigen-presenting cells (APCs), and incubated for several days to enable clonal expansion. Flow cytometry then quantifies the number of divided cells and daughter cell generations based on CFSE fluorescence levels.

Our in-house-formulated vaccine candidates utilize microparticles (MPs) and nanoparticles (NPs) to deliver the vaccine antigens efficiently. Here, we assessed the efficiency of our vaccine candidates in promoting T-cell proliferation in response to effective antigen presentation using the CFSE overlay assay and quantified using flow cytometry. The assay was optimized to quantify the T-cell proliferation for six days following antigen presentation. We tested our proof-of-concept particulate vaccine candidates that are formulated against bacterial pathogens *N*. *gonorrhoeae* strains CDC-F62, FA19, and FA1090 and viral pathogens measles, influenza A (H1N1), Zika, and canine coronavirus.

## 2. Materials and Methods

### 2.1. Materials

*Neisseria gonorrhoeae* strains FA1090 and FA19 were provided by Dr. Cynthia Cornelissen (Georgia State University, Atlanta, GA, USA), and CDC F62 by Prof. William M. Shafer (Emory University, Atlanta, GA, USA). The isolates were stored at −80 °C until further use. Dendritic cells (DC 2.4) and T lymphocytes (EL4) were purchased from the American Type Culture Collection (ATCC) (Manassas, VA, USA). Heat inactivated, NR-52286; influenza A virus, A/California/04/2009 (H1N1)pdm09; Zika virus strain PRVABC59 (1 × 106 PFU/mL) was kindly provided by Brandy Russell, Centers for Disease Control and Prevention (CDC), Colorado. Poly (D, L-lactide-co-glycolic acid) 75:25 (PLGA) (Resomer^®^ RG 752H) was purchased from Evonik Corporation. The adjuvants, Alhydrogel^®^ (Alum) and AddaVax™ (Analog of MF59), were purchased from Invivogen (San Diego, CA, USA). Bovine serum albumin (BSA) was purchased from Fisher Scientific (Waltham, MA, USA). The polymer poly (D, L-lactide-co-glycolide) 75:25 (PLGA) (Resomer^®^ RG 752 H) was procured from Evonik Corporation (Birmingham, AL, USA). Notably, the 5-(and 6)-Carboxyfluorescein diacetate succinimidyl ester Kit, CFDA SE Kit Biolegend (San Diego, CA, USA). Materials for cell culture—Dulbecco’s Modified Eagle medium (DMEM), fetal bovine serum (FBS), penicillin/streptomycin, and trypsin EDTA (ATCC^®^ TIB-71™) were purchased from American Type Culture Collection (ATCC, Manassas, VA, USA).

### 2.2. Methods

#### 2.2.1. Preparation of the Whole-Cell Inactivated *N. gonorrhoeae* Vaccine Antigens from the Strains CDC F62, FA19, and FA 1090

For the preparation of the microparticles, we followed the same protocol as established for the strain CDC F62 and described in our previous manuscripts [23,24]. Following the same protocol, we formulated vaccine microparticles for the new strains FA19 and FA1090. Briefly, the bacterial strains were grown on Gonococcal Base (GCB) agar containing defined Supplements I and II under 5.0% CO_2_ at 37 °C as described [3,4]. Palliated colonies were selected and further sub-cultured on a GCB agar plate overnight, which was used to inoculate two flasks containing 300 mL of GCB broth comprised of defined Supplement I and II with sodium bicarbonate (0.043%) in a 1000 mL sterile flask. The grown bacteria were then fixed using 10% formalin (*v*/*v*) and later harvested by centrifugation using sterile PBS at 5000× *g* for 15 min at 4 °C. The final collected pellets were pooled and saved as a very dense suspension at −80 ◦C till further use to formulate into vaccines.

#### 2.2.2. Preparation of Whole Cell Inactivated FA 1090, FA19, and CDC F62 *N. gonorrhoeae* Vaccine Microparticles and Adjuvant Microparticles and Characterization

Gonococci vaccine microparticles (Gc-MP) and adjuvant microparticles (Alum and AddaVax™) were synthesized using a protocol similar to a previously established protocol [10,23,24] within our laboratory, utilizing the Buchi Mini Spray Dryer B-290. Briefly, to initiate the process, we prepared a glutaraldehyde (25% aqueous solution, Fischer Scientific, Pittsburgh, PA, USA) cross-linked BSA solution to encapsulate the formalin-inactivated bacteria. For the generation of 100 mg of vaccine microparticles with a 10% antigen loading, a precise blend was prepared by combining 10 mg of formalin-fixed whole-cell *N. gonorrhoeae* (derived from a 2.5 mg/mL stock solution, 4 mL) with 90 mg of pre-cross-linked BSA and spray drying as described in the previous publications. Simultaneously, 100 mg of alum and 100 mg of AddaVax™ microparticles were prepared with a 10% loading (Supplementary Table S1). We further characterized the microparticles for particle size using dynamic light scattering (DLS) and surface charge (zeta potential) using a zeta sizer (Malvern Zetasizer Nano ZS, Westboroµgh, MA, USA), data previously published [10,23,24].

#### 2.2.3. Preparation of Particulate Vaccine Candidates for Viral Antigens Measles, Zika, Inactivated Canine Coronavirus (iCCoV), and H1N1 Flu Prototype

Measles vaccine microparticles were prepared and characterized as described in our previous publications [25,26]. Briefly, we utilized a 1% *w*/*v* solution of pre-crosslinked bovine serum albumin (BSA) with glutaraldehyde (200 µL glutaraldehyde per 1 g of BSA). The excess of glutaraldehyde was neutralized with sodium bisulfite. Measles vaccine antigen (Edmonston–Zagreb strain) was added to this (5% *w*/*w*), and the formulation was spray dried through Buchi mini spray dryer B-290 through a 0.5-mm nozzle (nozzle temperature −5 °C) at a flow rate of 20 mL/h to obtain the measles vaccine-loaded microparticles [27].

Zika virus vaccine antigen was cultured, inactivated, and purified using the same method as described in our previously published protocol [12]. We cultured Zika using VERO cells, inactivated, and harvested the virus using 0.03% beta-propiolactone (BPL) (Fischer Scientific, Hampton, NH, USA) for 48 h. The inactivated virus was purified using Centricon^®^ Plus-70 centrifugal filters (Millipore Corporation, Burlington, MA, USA), used as the vaccine antigen, and further encapsulated into PLGA-based vaccine microparticles (MP). The Zika virus-loaded PLGA microparticulate vaccine formulation utilized a double emulsion solvent evaporation method as described previously [12]. Briefly, the antigen was emulsified in a 2% polymer solution (PLGA in dichloromethane (DCM)) using the Omni THQ probe homogenizer (Kennesaw, GA, USA) to obtain the primary emulsion (Supplementary Table S1). A double emulsion was obtained as the primary emulsion was further emulsified with 0.1% polyvinyl alcohol (PVA, MW 30,000–70,000, Sigma–Aldrich, St. Louis, MI, USA) solution and homogenized using Nano DeBEE high-pressure homogenizer. DCM was evaporated and the emulsion was ultracentrifuged to concentrate the MP, which was freeze-dried with trehalose as the cryoprotectant using a Labconco™ FreeZone Triad benchtop freeze dryer. Adjuvant MP encapsulating Addavax^TM^ MPs and Alhydrogel^®^ MP were formulated following a similar method.

Inactivated influenza A virus vaccine microparticles were formulated using a previously established double emulsion method protocol described in [28]. Here, heat-inactivated influenza virus strain A/California/04/2009 (H1N1)pdm09 (1%) in a pH 7.4 phosphate buffer was added to PLGA in DCM solution (2% *w*/*v*) and probe homogenized at 17,000 RPM using a 30 s on/off cycle for 2 min (primary emulsion). Next, the primary emulsion was probe homogenized with the PVA solution in deionized water (0.1% *w*/*v*) for 2 min at 17,000 RPM (double emulsion). The final emulsion was stirred at 500 RPM for 5 h to remove the residual DCM via solvent evaporation. The excess PVA was removed by washing with deionized water, followed by centrifugation at 17,000 RPM for 10 min.

Inactivated canine coronavirus (iCCoV) vaccine MPs and Addavax^TM^ MPs were formulated using a previously established formulation protocol as published [28]. The vaccine antigen (1% loading) in phosphate buffer (pH 7.4) was added to a 2% PLGA solution in DCM and homogenized for 2 min at 17,000 RPM to form the primary emulsion. This primary emulsion was then added to a 0.1% PVA solution and homogenized again at 17,000 RPM for 2 min to create the double emulsion. Upon evaporating DCM, the excess PVA was washed and concentrated into a pellet. The pellet was resuspended in trehalose solution and further lyophilized.

#### 2.2.4. Imaging of Antigen Presentation by Stimulated DCs to the Overlaid Naïve T-Cells

We utilized the Agilent BioTek LionHeart Live Cell Imager to capture the imaging of cell-to-cell contact of the innate and adaptive immune cells in the DC and naïve T-cell overlay models. DCs were plated in a 6-well plate, stimulated with indocyanine green (ICG)-labeled vaccine nanoparticles, and incubated for 4 h at 37 °C. The DCs were observed to be activated and stimulated by the exposure to the BSA NPs, and internalization of the ICG-labeled NPs was evidenced. On the same 6-well plate, we introduced DAPI-stained naïve T-cells, which are non-adherent in nature, and observed the naïve T-cells interacting with the stimulated DCs and imaged the process of antigen presentation to the T-cells. Upon antigen presentation, the T-cells were observed to move away from the interaction, and subsequent T-cell division was imaged as shown below.

#### 2.2.5. Assessment of Effective Antigen Presentation In Vitro

##### Pulsing DCs (APCs) with Various Vaccine Treatments

Dendritic cells (DC 2.0) were adjusted to 0.12 × 10^6^ cells/well in a 48-well plate. In this setup, the groups were divided into control and treatment groups. The control groups included unstained blank T-cells only and CFSE-stained cells only with no treatment. Both control groups were not pulsed with the vaccine particles. The treatment group wells were DCs treated with previously diluted *N. gonorrhoeae*, strains CDC F62, FA19, and FA1090 vaccine microparticles along with albumin-based Alum and Addavax^®^ adjuvants and viral vaccine candidates against H1N1, measles, and Zika virus loaded on PLGA nanoparticles along with Alhydrogel^®^ (Alum) and AddaVax™ (Analog of MF59) PLGA particles. For the dose-dependent response study, concentrations ranged from 200 µg–40 µg per well (antigen-loading table provided in Supplementary Table S1). We also established blank treatment groups where wells were treated with blank BSA MPs and blank PLGA NPs. After adding the particles, the plates were incubated for 24 h at 5% CO_2_ and 37 °C.

##### Preparing CFSE-Stained Naïve T-Cells

We utilized an EL4 cell line from the American Type Culture Collection (ATCC) (Manassas, VA, USA). The CFSE dye kit was utilized per the company instructions below (details also included in the CellTrace™ CFSE kit, Invitrogen, ref. C34554). Briefly, the vial of lyophilized reagent was centrifuged in a microcentrifuge to ensure that the reagent settled at the bottom of the vial. The stock solution was prepared by reconstituting 1 vial of lyophilized CFSE dye in 36 µL of dimethyl sulfoxide (DMSO) to create a 5 mM solution. A 5 µM working solution was then generated by diluting 1 µL of the 5 mM CFSE stock solution in 1 mL of phosphate-buffered saline (PBS) for every 1 mL of the cell suspension or at an optimal working concentration as determined by titration. The T-cells were spun down and resuspended at 10–100 × 10^6^ cells/mL in the CFSE working solution. The incubation of cells occurred for 20 min at either room temperature or 37 °C, with protection from light. The staining was quenched by adding five times the original staining volume of DMEM medium containing 10% fetal bovine serum (FBS). After pelleting and resuspending the cells in a pre-warmed cell culture medium, the cells were incubated for 10 more minutes. Following incubation, the CFSE-labeled cells were considered ready for downstream applications, here overlay assay.

##### Overlaying the Antigen-Presenting Cells (DCs) with CFSE-Stained Naïve T-Cells for the Antigen Presentation Assessment

The DCs and particles were incubated for 24 h after the vaccine particle addition. After 24 h, the excessive media and particles were removed from the wells by aspirating old media and washing the wells gently with complete DMEM media. To these pulsed DCs, T-cells stained with the CFSE dye as described in Section 2.2.5 were introduced into the wells at a concentration of DCs:T-cells 1:5 ratio. Every well was incubated with 0.6 × 10^6^ cells/well of CFSE-stained T-cells. Upon overlaying the stained T-cells over the activated DCs, the plates were gently placed in the incubator and incubated for 6 days.

##### Flow Cytometry Analysis

On days 0, 1, 2, 4, and 6, T-cells from the designated wells were collected into 2 mL Eppendorf tubes, followed by centrifugation at a speed of 1100 RPM for 7 min at 4 °C temperature. The cell pellet was washed three times with sterile PBS to remove excess dye. After the final wash, the pellet was resuspended in 1 mL of sterile PBS, filtered using a 70 µm cell strainer, kept on ice, protected from light, and analyzed using a BD Accuri C6 flow cytometer (Becton, Dickinson, and Company, San Diego, CA, USA). The live cell population was gated with unstained cells. For each sample, 10,000 events were recorded in the gate for the live lymphocyte population, and cell division in live cells was quantitatively assessed at a 517 nm wavelength. To establish the assay’s versatility, we quantified T-cell proliferation in terms of both mean FITC-A expression-direct comparison of FITC comparison and percent T-cell proliferation-isolating % T-cells proliferated as days progressed by establishing gates along the *X*-axis of histograms representing FITC-A expression.

#### 2.2.6. Dose-Response Analysis of T-Cell Proliferation in Response to Various Concentrations of the Adjuvanted Vaccine Particles

We conducted dose-response studies for all antigen-loaded vaccine candidates to assess variations in T-cell proliferation using the overlay assay. The doses of antigen-loaded particles that were tested ranged from 200 µg, 160 µg, 120 µg, 80 µg and 40 µg per well for viral and bacterial vaccine candidates. We conducted this study following the same procedures for all the overlay assays, as explained in Section 2.2.5.

#### 2.2.7. Statistical Analysis

All experiments were performed in triplicate unless otherwise stated. For statistical analysis, if the data were normally distributed, then ordinary one-way ANOVA or Brown–Forsythe and Welch ANOVA tests were used for independent groups, and two-way ANOVA was used for dependent groups. If the data were not normally distributed, a Kruskal–Wallis test was used. A post-hoc Tukey test was used to compare means, and a post-hoc Dunnett test was used to compare means to the control. Mean values ± SEM and *p*-values were calculated individually for all experiments using GraphPad Prism 8.4.3 software (GraphPad Software, San Diego, CA, USA).

## 3. Results

### 3.1. Imaging of Antigen Presentation by the DCs during Antigen Presentation to T-Cells and Subsequent T-Cell Division

**Figure 1:**
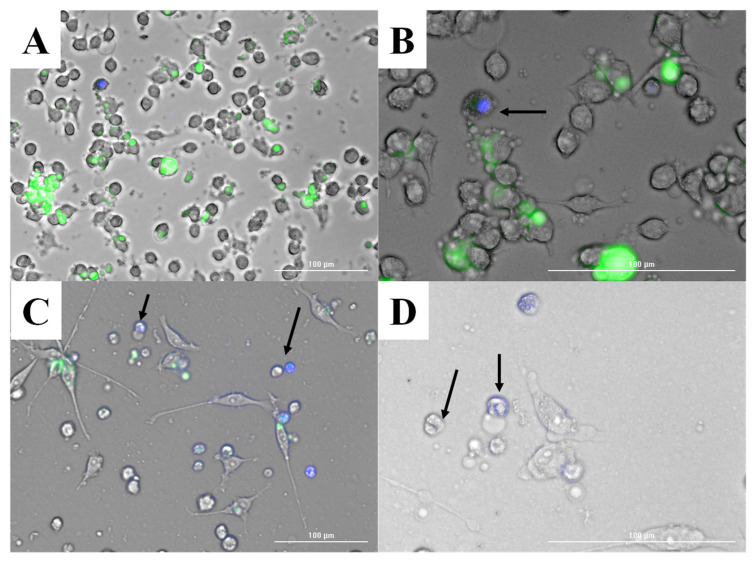
Live cell imaging of DAPI-stained naïve T-cells interacting with activated dendritic cells. (**A**): Overview of the culture showing DAPI-stained T-cells (blue) interacting with activated dendritic cells that are stimulated with ICG-coated BSA MPs (green) across the field. Scale bar: 100 µm. (**B**): Close-up view highlighting a DAPI-stained T-cell engaging with a dendritic cell, indicated by the black arrow. Scale bar: 100 µm. (**C**): Magnified image displaying multiple T-cells in the process of interacting with dendritic cells. Black arrows indicate T-cells undergoing division. Scale bar: 100 µm. (**D**): Detailed image of T-cells post-division, as indicated by black arrows, continuing their interaction with dendritic cells. Scale bar: 100 µm.

Upon stimulating the DCs with ICG-coated BSA MPs, the DCs internalized in the MPs in 4 h. The interaction of DAPI-stained naïve T-cells laid over the activated DCs for antigen presentation is shown in Figure 1A,B. The DAPI-stained T-cells moved away from the site of cell-to-cell contact interaction, where antigen presentation took place, and proliferated into daughter T-cells (Figure 1C,D). The imaging of the antigen presentation and subsequent T-cell division confirmed the ability of the vaccine microparticle-loaded DCs to induce T-cell proliferation.

### 3.2. Isolation and Quantification of Proliferated T-Cells Using Flow Cytometry

**Figure 2:**
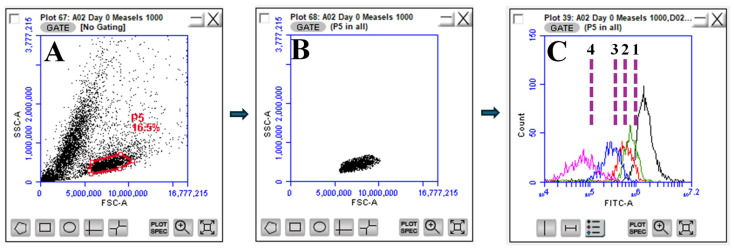
Representative flow cytometry data of T-lymphocyte profiling. (**A**). Gating strategy for separating T lymphocytes from the forward scattering vs. side scattering plot. T-cells were gated, capturing 16.5% of the total population of the scatter plot. (**B**). Singlets are shown on the forward scattering a vs. forward scattering height plot from the T lymphocyte gating. (**C**). Histograms gated 1, 2, 3, and 4 according to daughter T-cell proliferation over time intervals: 0–1, 1–2, 2–4, and 4–6 days, respectively. Gates were established in accordance with the proliferation pattern of bacterial and viral-based vaccine candidates. Gates were left unchanged for the corresponding blank MP/NP groups.

The CFSE-overlay analysis in response to nanoparticulate vaccine candidates at 200 µg per well dose was performed using flow cytometry. Proliferated T-cells or daughter cell populations were quantified based on the percentage of T-cells proliferated from days 0–6 in response to nanoparticulate vaccine candidates pulsed in DCs and subsequent antigen presentation to the T-cells. From the flow cytometry analysis, live T lymphocytes were isolated by applying gating strategies outlined in Figure 2 and represented in histograms to visualize the propagation of daughter T-cells from parent T-cells, reflecting T-cell clonal expansion over the days of exposure to pulsed DCs. We established gates along the *X*-axis, representing FITC-A expression, to isolate the proliferated live T-cell daughter populations as the days progressed. Figure 2C demonstrates the gating strategy in the measles vaccine NPs + adjuvants treated groups. The gates established for all other groups are summarized in Supplementary Table S2. We quantified the percentage of cell proliferation for measles vaccine NPs + adjuvants, Zika vaccine NPs + adjuvants, inactivated H1N1 flu vaccine + adjuvants, canine coronavirus NPs + adjuvants, N. *gonorrhoeae* strains CDC F62, FA19, and FA1090 vaccine MPs + adjuvants, blank PLGA NPs, and blank BSA MPs and reported the results in Table 1.

**Figure 3:**
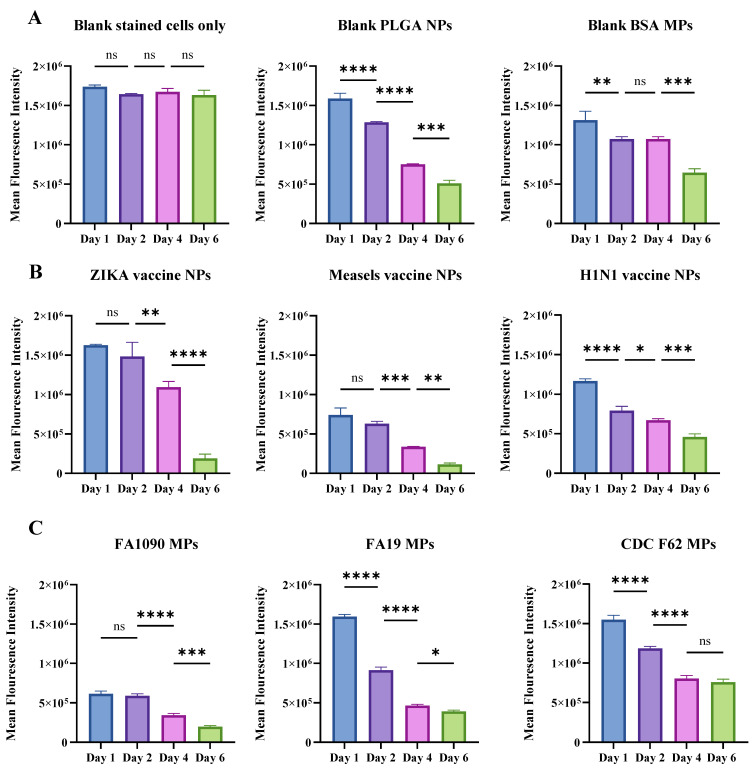
Quantitative comparisons of CFSE (FITC-A filter) expressions due to T-cells proliferated as days passed. CFSE is expressed by the proliferating T-cells in response to antigen presentation by the DCs upon stimulation by various treatment groups. (**A**). Comparison of all blank groups involved in the experiment, including blank CFSE-stained T-cells only, blank BSA MPs, and blank PLGA NPs. (**B**). comparison of all viral antigen-based vaccine candidates. (**C**). comparison of all bacterial antigen-based vaccine candidates. All treatments are at 200 µg per well dose. Data are expressed as mean ± SEM, ordinary one-way ANOVA test, post-hoc Tukey’s multiple comparison test. ns, non-significant, * *p* ≤ 0.05, ** *p* ≤ 0.01, *** *p* ≤ 0.001, **** *p* ≤ 0.0001.

The proliferation of daughter T-cells was analyzed across different vaccine treatments and control groups over a span of six days and summarized in Table 1. The mean FITC-A expression of the proliferated daughter T-cells was compared and represented the trends in Figure 3. The percent proliferation of the daughter T-cells was calculated according to the gates established and represented in Table 1. Measles vaccine NPs consistently induced high proliferation rates, maintaining over 77% from Day 0 through Day 6, with a peak of 91.08% on Day 2. Similarly, H1N1 flu prototype vaccine NPs combined with adjuvants demonstrated a strong proliferative response, starting at 52.5% on Day 0 and sustaining above 84% from Day 2 to Day 6. Zika vaccine NPs with adjuvants showed the highest proliferation (98.25%) on Day 4, maintaining robust activity through Day 6 (85.21%). The canine coronavirus vaccine NPs with adjuvants had moderate proliferation initially (43.28%) that increased substantially by Day 2 (72.45%) but reduced to 61.74% by Day 6. *N. gonorrhoeae* FA1090 vaccine MPs and *N. gonorrhoeae* CDC F62 vaccine NPs with adjuvants both demonstrated substantial and sustained T-cell proliferation, peaking on Day 2 (80.25% and 67.7%, respectively) and maintaining high levels through Day 6. In contrast, the CFSE-stained T-cells alone showed a marked initial proliferation (60.16%) that sharply declined by Day 1 and ceased entirely by Day 2. Blank PLGA NPs exhibited sustained proliferation peaking on Day 2 (88.98%), followed by a notable decline by Day 6 (33.95%). Blank BSA MPs with adjuvants followed a similar pattern, peaking on Day 2 (78.65%) and maintaining moderate levels by Day 6 (61.54%), representing a significantly lower proliferation than vaccine BSA MPs. These results indicate that vaccine nanoparticles combined with adjuvants can significantly enhance T-cell proliferation over an extended period compared to CFSE-stained T-cells alone and blank nanoparticle controls. Viral vaccine formulations (measles, H1N1, Zika, and canine coronavirus) induced higher and more sustained proliferation compared to bacterial vaccine formulations (*N. gonorrhoeae* FA1090, FA19, and CDC F62). This suggests a potentially stronger and longer-lasting immune response elicited by viral vaccines compared to bacterial vaccines, highlighting the variability in promoting robust immune responses by the antigenic epitopes.

### 3.3. Dose-Dependent Response Study for Various Antigen-Loaded Particulate Vaccine Candidates

**Figure 4:**
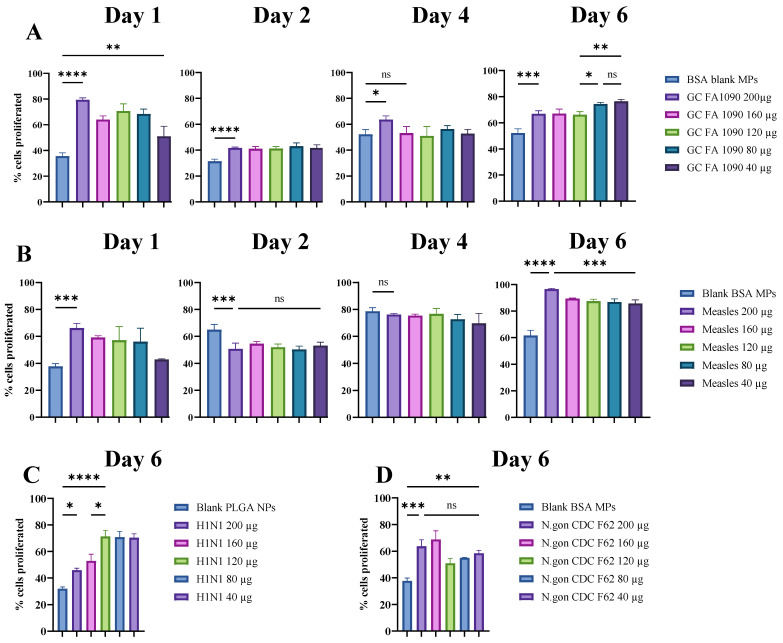
Dose-dependent study results quantifying T-cell proliferation against various concentrations of vaccine candidates. (**A**). T-cell proliferation analysis measured on days 1, 2, 4, and 6 when treated with vaccine candidate against *N. gonorrhoeae* strain FA1090 at concentrations of 200 µg, 160 µg, 120 µg, 80 µg, and 40 µg vaccine MPs per well. (**B**). T-cell proliferation analysis was measured on days 1, 2, 4, and 6 when treated with a vaccine candidate against the measles virus at concentrations of 200 µg, 160 µg, 120 µg, 80 µg, and 40 µg vaccine NPs per well. (**C**,**D**). T-cell proliferation trends quantified in response to H1N1 virus particle vaccine candidate and *N. gonorrhoeae* strain CDC F62 bacterial particle vaccine candidates on day 6. Both were tested at concentrations of 200 µg, 160 µg, 120 µg, 80 µg, and 40 µg per well on day 6. Data are expressed as mean ± SEM, one-way ANOVA, post hoc Tukey’s multiple comparisons test; ns, non-significant, * *p* ≤ 0.05, ** *p* ≤ 0.01, *** *p* ≤ 0.001, **** *p* ≤ 0.0001.

Dose–response studies have been conducted where the proliferation of the T-cells against various doses of the viral and bacterial vaccine candidates was measured. We quantified the T-cell responses that occurred as a result of antigen presentation by the dendritic cells when exposed to several vaccine candidates at concentrations of 200 µg, 160 µg, 120 µg, 80 µg, and 40 µg and monitored on days 1, 2, 4, and 6 that followed. This study revealed significant dose-dependent T-cell proliferation in response to *N. gonorrhoeae* FA1090, *N. gonorrhoeae* FA19, and *N. gonorrhoeae* CDC F62 bacterial strains; Zika, H1N1, and measles viral vaccine candidates. The results for measles and *N. gonorrhoeae* FA1090 are shown in Figure 4A, while Figure 4B shows the proliferation trend on day 6 for *N. gonorrhoeae* CDC F62 and H1N1 flu prototype vaccine candidates in Figure 4C,D. The proliferation results for Zika and *N. gonorrhoeae* FA19 strain vaccine candidates are shown in Table 2.

This study revealed significant dose-dependent T-cell proliferation in response to the Zika, *Neisseria gonorrhoeae*, FA1090, FA19, and CDC F62, H1N1, and measles vaccine candidates. For the *N. gonorrhoeae* FA19 vaccine, higher doses (200 µg and 160 µg MPs) induced the most significant proliferation by day 6, followed closely by the 120 µg MPs. Lower doses (80 µg and 40 µg MPs) also showed significant proliferation, demonstrating a dose-dependent response as shown in Table 2. The *N. gonorrhoeae* CDC F62 vaccine exhibited similar trends, with the highest proliferation observed at 160 µg, followed by 200 µg, and moderate responses at lower doses (40 µg, 80 µg, and 120 µg), as shown in Figure 4D. However, FA1090 exhibited more potent proliferation at lower doses (80 and 40 µg per well) compared to the higher doses, as shown in Figure 4A. In contrast, the H1N1 vaccine showed the highest T-cell proliferation at the 120 µg dose by day 6, with slightly lower but still substantial responses at 80 µg and 40 µg. Higher doses (160 µg and 200 µg) resulted in moderate proliferation, indicating an optimal dose range around 120 µg for maximal response, as shown in Figure 4C. For the measles vaccine, the 200 µg dose induced the highest T-cell proliferation by day 6, with the 160 µg, 120 µg, and 80 µg doses also showing substantial responses and the 40 µg dose exhibiting significant proliferation, as demonstrated in Figure 4B. For the Zika vaccine, the highest doses (200 µg MPs and 160 µg NPs) showed the most robust proliferation by day 6. The 120 µg NPs also demonstrated substantial proliferation, while lower doses (80 µg and 40 µg NPs) showed moderate responses, indicating a clear trend of increased proliferation with higher doses (Table 2). The control groups with blank PLGA NPs and blank BSA MPs showed moderate proliferation levels, with a peak on day 4 and a weak increase by day 6 compared to the antigen-loaded vaccine candidates. The findings on day 6 highlight the importance of dose optimization, with higher doses generally resulting in stronger immune responses for the viral vaccine candidates tested and a mixed trend in the bacterial vaccine candidates. Control groups confirmed that the observed proliferative responses were due to the vaccine antigens rather than the delivery vehicles, underscoring the efficacy of the vaccines at specific dose ranges. These results emphasize the need for careful dose selection to achieve maximal immunogenicity in vaccine development.

## 4. Discussion

In this study, the optimized CFSE-stained cell-to-cell contact assay to quantify T-cell proliferation in response to various particulate vaccine candidates when overlaid with the antigen-presenting cells is reported. This in vitro assay has been optimized for multiple variables, including cell density per well, the ratio of APCs to T-cells per well, the number of particles used to pulse APCs in the well, the ratio of the concentration of antigen-loaded vaccine candidates to adjuvant-loaded microparticles, and the number of incubation days needed after antigen presentation to record the optimal T-cell proliferation. The influence of these parameters was examined, and the variations in antigen presentation were recorded with respect to antigen type, carrier variation, and dose variation of the vaccine candidates. In our studies, we tested a 1:1, 1:5, and 1:10 ratio of DCs to T cells per well. The 1:1 ratio did not facilitate the antigen presentation as days progressed as much as the 1:5 ratio as we quantified the subsequent T cell proliferation. The 1:10 ratio conducted led to rapid T-cell proliferation; however, we experienced a loss of viable cells due to overcrowding of the cells per well, which could be due to a simple lack of nutrition as the setup could not accommodate the volume of cells. We found that a ratio of 1:5 APCs to T cells led to an optimal exposure of the stimulated DCs to the T-cells for successful antigen presentation while maintaining viable conditions for the CFSE-stained T-cells over the days of analysis. We evaluated the importance of the addition of adjuvants to vaccine candidates in promoting antigen presentation. The effects of Alhydrogel^®^ (Alum) and AddaVax™ (analog of MF59) and CpG adjuvants that are encapsulated into nanoparticles in-house, individually and in combination, are tested. The results indicated the addition of all three adjuvant particles as a combination did not provide comparatively significant robust T-cell proliferation compared to the combination of Alhydrogel^®^ (Alum) and AddaVax™ (analog of MF59). Moreover, adding all three adjuvant particles combined with the vaccine candidates led to overcrowding in the well and cell death. Therefore, we limited the adjuvant addition to only Alhydrogel^®^ (Alum) and AddaVax™ (analog of MF59) nanoparticles. We accounted for the amount of vaccine antigen added per well by considering the loading of the nanoparticle formulations with the inactivated antigens. The viral nanoparticle vaccine candidates comprise 2% loading, whereas the bacterial microparticle vaccine candidates comprise 20% loading, as shown in Supplementary Table S1. In total, 200 µg of the vaccine candidate microparticles for both bacterial and viral vaccine formulations were added, which equaled a final 40 µg of bacterial vaccine antigen and 4 µg of viral vaccine antigen were added per well. The results showed that bacterial vaccine candidates showed significantly higher T-cell proliferation at lower concentrations of vaccine particles per well, whereas higher concentrations were observed for viral vaccine candidates. This observation for the *N. gonorrhoeae* bacterial vaccine candidate can be due to the characteristic of *N. gonorrhoeae* in evading the immune system [29]. *N. gonorrhoeae* antigens in suspension form can be lethal to immune cells such as the APCs; thus, at higher concentrations, as the inactivated vaccine antigens are released from the encapsulated microparticles, it might induce CD95 and death pathways in immune cells, leading to diminished antigen presentation and subsequent T-cell proliferation [30,31]. However, we observed variation in responses to higher concentrations for various strains of the *N. gonorrhoeae* vaccine candidates. While this argument might hold strong in the case of the FA1090 strain vaccine candidate, the other two strains, FA19 and CDC F62, showed improved T-cell proliferation at higher concentrations of the vaccine candidate per well.

Varied responses within the viral groups of antigens were also observed. The measles vaccine candidate showed the highest initial proliferation and sustained high levels of proliferation throughout the days of analysis. In contrast, vaccine candidates such as Zika showed an initial decrease followed by a substantial increase in T-cell proliferation by day 6, suggesting the potential of Zika vaccine formulation in sustaining antigen presentation over time, particularly at higher concentrations. This observation is critical in understanding the role of pathogen antigen type in producing varying T-cell proliferation responses, a quick initial and sustaining response or a delayed but robust and sustained response over time, and the dose of the vaccine candidates in accomplishing effective antigen presentation and subsequent T-cell proliferation. The overlay assay also provided us with a platform to understand the ability of different carriers to facilitate antigen presentation. Here, we tested a BSA-based microparticulate system and a PLGA-based nanoparticle system as vaccine carriers. Compared to the no particle treatment, the CFSE-stained T-cells only group that showed no proliferation of T-cells past day 2, the blank BSA microparticle and blank PLGA nanoparticle treated groups showed T-cell proliferation throughout the days of analysis. Although blank BSA microparticulate and blank PLGA nanoparticulate groups elicited T-cell proliferation through days 1, 2, and 4, by day 6, there was a significant reduction in proliferation as compared to the vaccine antigen-loaded BSA and PLGA treatment groups. This pattern suggests that while blank particles can initially stimulate T-cell proliferation, the effect diminishes over time, likely due to the lack of specific antigenic stimulation, highlighting the importance of the presence of antigens and adjuvants for enhanced antigen presentation. These variations highlight the vaccine candidates’ differential efficacy and kinetics in inducing T-cell proliferation, emphasizing the potential for selected formulations to elicit stronger and more durable immune responses following optimal antigen presentation. Obtaining this information through this rapid, high-throughput assay prior to testing these formulations in in vivo studies added validation to the formulation and quality control process of vaccine development.

Assessing effective antigen presentation using overlay assays has become integral to preclinical testing of vaccine formulations, particularly in early-stage evaluations following initial in vitro assays confirming antigen stability and integrity [32]. These assays enable high-throughput screening and real-time analysis, generating quantitative data on immune parameters such as effective antigen presentation, T-cell repertoire assessment, T-cell exhaustion [5], cytokine production [33], B-cell differentiation assessment, detection of autoimmune reactions, cross-reactivity, affinity and avidity of antigen interactions, and adjuvant effects [34]. Overlay assays provide a direct assessment of binding interactions and subsequent signal transduction events [35]. This reveals the mechanisms by which vaccine antigens activate or modulate critical immune pathways [36], including changes in phosphorylation patterns and evaluation of cellular responses such as cytokine production and proliferation [37]. Integrating overlay assay data with bioinformatics and systems biology enhances the understanding of immune signaling networks [38], supporting the development of personalized vaccination strategies and reducing complications post-vaccination against infectious diseases [39] and cancers [40]. Overlay assays can help identify promising vaccine formulations early and ensure that only the most effective and safe candidates proceed to more resource-intensive animal testing and clinical trials.

However, further longitudinal studies are required to confirm these findings and understand their long-term implications for a vaccine’s success. Immunological assays such as neutralizing antibody titers and ELISAs, along with functional assays such as virus neutralization and opsonophagocytic assays, provide direct measures of immune effectiveness [41]. Functional studies are needed to assess T-cell functionality, including vaccine antigen recognition, cytokine production, and comparative efficacy in animal models [42]. System biology approaches offer comprehensive insights into immune responses at the genomic, transcriptomic, proteomic, and metabolomic levels [43]. Human clinical trials across phases I to III are essential to ensure safety, immunogenicity, and efficacy, with long-term follow-up for sustained immunity and adverse effects [44]. Extended time-point analysis of T-cell proliferation to ensure sustainability, evaluating memory T-cell formation for long-lasting immunity [45], and evaluating booster dose effects help optimize vaccination schedules [46,47]. Additionally, identifying correlates of protection (CoPs) can significantly accelerate vaccine development by providing early indicators of vaccine efficacy [48,49,50]. Epidemiological and longitudinal cohort studies link immune responses to protection in large populations, offering valuable insights into the duration and robustness of vaccine-induced immunity [51,52,53].

Although overlay assays prove to be highly beneficial in vaccine preclinical development, there are shortcomings in optimizing the assay as it requires sophisticated equipment and technical expertise, given the complexity. Given the fact that in vitro assays cannot replicate the complexity of the in vivo immune environment, including tissue-specific responses and systemic signals, predictive accuracy can be limited in terms of the correlation of outcomes [54,55,56]. They often involve isolated immune cells, lacking the full complement of immune system components present in vivo, potentially resulting in incomplete or skewed data. Another factor contributing to the error in the assays is the variability in cell culture conditions, and operator technique can affect reproducibility, posing challenges for consistent results. Although faster and less resource-intensive than animal studies, overlay assays still require significant time and cost for optimization and execution, in our case, a total of 8 days. They often involve isolated immune cells, lacking the full complement of immune system components in vivo, potentially resulting in incomplete or skewed data. Some reliable techniques to address these shortcomings are standardizing protocols, employing advanced equipment for automation, and providing comprehensive training that can improve reproducibility and consistency [57]. Incorporating 3D cell cultures and organ-on-a-chip models can enhance physiological relevance from in vitro overlay assays to in vivo findings [58,59,60,61,62].

Quality control during vaccine formulation development is critical to ensure that vaccines effectively induce the desired immune response while minimizing adverse effects [63]. This rigorous process includes various longitudinal studies such as antigen characterization, adjuvant testing, immunogenicity testing through preclinical and clinical trials, potency and stability assays, and sterility checks, along with maintaining batch-to-batch consistency and conducting post-market surveillance for monitoring real-world effectiveness and safety [15]. Despite these comprehensive measures, vaccines can still have potential side effects, as documented in recent years, demanding more vigorous screening for safer and personalized vaccination strategies. Aside from common mild side effects such as pain at the injection site, fever, and fatigue, there have also been reports of more severe but rare adverse effects, including myocarditis and pericarditis associated with mRNA COVID-19 vaccines, thrombosis with thrombocytopenia syndrome (TTS) linked to adenovirus vector vaccines [64], Myasthenia gravis (MG) [63], and Guillain–Barré syndrome (GBS) observed in some vaccine recipients. Additionally, there are concerns about autoimmune and inflammatory responses potentially induced by adjuvants, known as autoimmune/inflammatory syndrome induced by adjuvants (ASIA) [65,66]. Mechanisms such as molecular mimicry, where vaccine antigens resemble self-antigens, can potentially trigger autoimmune responses [67]. This phenomenon may lead to the activation of auto-reactive T-cells, causing conditions such as lupus [68] or rheumatoid arthritis [69]. The temporal relationship between vaccination and the onset of autoimmune conditions necessitates careful monitoring and investigation to identify any causative links [64]. Adverse events following immunization (AEFI) highlight the importance of robust surveillance systems to detect and manage these events promptly [70]. The potential for neurological side effects, such as Guillain–Barré syndrome and encephalitis [71], underscores the need for ongoing vigilance and research [66]. Identifying biomarkers that can predict these adverse reactions is essential for developing personalized vaccine strategies that minimize risks for susceptible individuals.

A recent deep multi-omics study identified key risk factors for post-acute sequelae of COVID-19 (PASC) [72], including type 2 diabetes, the presence of SARS-CoV-2 RNA in the blood (RNAemia), Epstein–Barr virus (EBV) viremia, and specific autoantibodies [72]. These factors were found to correlate with the likelihood of developing chronic symptoms and unique immune responses in patients with gastrointestinal post-acute sequelae of SARS-CoV-2 (PASC), including specific dynamics of SARS-CoV-2-specific and cytomegalovirus (CMV)-specific CD8+ T-cells during recovery, highlighting the importance of early detection and personalized vaccination strategies [73]. Another identification is the development of Guillain–Barre syndrome and auto-antibodies in response to vaccination against pathogenic bacteria, *Campylobacter jejuni* (*C. jejuni*) [74]. It is now established that *C. jejuni* possesses molecular mimicry, resembling glycolipids found on human peripheral nerves [75]. Thus, when vaccinated, the immune system produces antibodies against the surface antigens of *C. jejuni*, which can be cross-reactive towards the human nervous system glycolipids, leading to nerve damage and causing neurological disorders [76]. In addition, certain individual HLA alleles are more prevalent in individuals who develop GBS after *C. jejuni* infection, suggesting a genetic basis for susceptibility [77]. The specific structure of pathogenic lipo-oligosaccharides (LOS) in *C. jejuni* can activate the immune system via Toll-like receptor (TLR)-4 signaling [78], contributing to the severity [76]. In contrast, deep omics studies can also reveal individuals with autoimmune diseases such as Sjögren’s syndrome possess no greater severity in experiencing adverse effects of the COVID-19 vaccine compared to healthy individuals [79]. These findings highlight the importance of molecular mimicry and dysregulated immune responses in individuals, demanding early screening and personalized vaccination strategies [79].

## 5. Conclusions

In conclusion, we successfully optimized a cell-to-cell contact or T-cell-APC overlay assay to quantify the T-cell proliferation in vitro. We evaluated our in-house formulated particulate vaccine candidates for their ability to stimulate antigen presentation by the APCs, followed by T-cell stimulation and a robust clonal expansion against a specific pathogen. We utilized the CFSE assay to quantify the T-cell daughter populations that were proliferating as a response to the vaccine particle stimulation using flow cytometry. Our data supports this optimized protocol to test microparticulate vaccine formulations containing various bacterial or viral antigens’ ability to induce effective antigen presentation and naive T-cell proliferation. Utilizing this optimized assay, we screen our future vaccine formulations to identify promising vaccine candidates early, ensuring that only the most effective and safe candidates proceed to more resource-intensive animal testing and clinical trials.

## Figures and Tables

**Table 1:** Proliferation of T-cell daughter cells in response to vaccine candidate treatments over time.

Treatment Groups	Percent Daughter T-Cells Proliferated (%) beyond the Established Gates			
	Day 0–Day 1	Day 1–Day 2	Day 2–Day 4	Day 4–Day 6
CFSE-stained T-cells only	60.2 ± 1.7%	11.5 ±14.1%	0%	0%
Blank PLGA NPs	51.4 ±6.5%	56.6 ±4.4%	89.0 ± 6.7%	34.0 ±13.4%
Measles Vaccine NPs + Adjuvants	77.3 ± 6.8%	65.5 ± 8.1%	91.1 ± 0.2%	87.5 ± 9.0%
H1N1 Flu Vaccine NPs + Adjuvants	52.5 ± 2.6%	71.8 ± 15.1%	84.6 ±6.6%	86.0 ±3.8%
Zika Vaccine NPs + Adjuvants	55.9 ±1.5%	74.1 ±7.0%	98.3 ±3.1%	85.2 ± 9.2%
Canine coronavirus vaccine NPs + Adjuvants	43.3 ± 0.38	72.5 ± 3.68%	52.0 ±0.7%	61.7 ± 8.8%
Blank BSA MPs	37.7 ± 5.5%	50.7 ± 8.5%	78.7 ± 3.4%	31.5 ±6.4%
*N. gonorrhoeae* FA1090 Vaccine MPs + Adjuvants	45.6 ±6.4%	68.8 ± 4.5%	80.3 ± 3%	85.0 ± 3.5%
*N. gonorrhoeae* FA19 Vaccine MPs + Adjuvants	29.3 ± 2.6%	46.7 ± 4.3%	66.9 ± 4%	86.9 ± 9.1%
*N. gonorrhoeae* CDC F62 Vaccine NPs + Adjuvants	48.4 ± 13.7%	67.5 ± 4.4%	67.7 ± 5.4%	79.0 ± 15.6%

**Table 2:** Dose-dependent proliferation measurements of T-cells in response to Zika vaccine MPs and *N. gonorrhoeae* FA19 MPs with adjuvants on days 1, 2, 4, and 6.

Treatment Groups	Percent T-Cells Proliferated as Days Progressed (%)			
	Day 1	Day 2	Day 4	Day 6
PLGA Blank NPs	54 ± 2.6%	12 ± 4.2%	29 ± 0.6%	31 ± 7.4%
Zika vaccine MPs 200 µg + adjuvants	57 ± 1.7%	9 ± 0.8%	26 ± 2.1%	83 ± 8.9%
Zika vaccine NPs 160 µg + adjuvants	56 ± 1.5%	18 ± 1.2%	13 ± 9.3%	81 ± 3.2%
Zika vaccine NPs 120 µg + adjuvants	54 ± 2.0%	12 ± 2.2%	19 ± 13.3%	73 ± 5.0%
Zika vaccine NPs 80 µg + adjuvants	51 ± 2.5%	21 ± 5.6%	11 ± 12.8%	65 ± 6.4%
Zika vaccine NPs 40 µg + adjuvants	52 ± 5.7%	29 ± 1.7%	0%	67 ± 1.3%
BSA Blank MPs	52 ± 2.1%	21 ± 1.8%	10 ± 3.1%	52 ± 3.2%
GC FA19 vaccine MPs 200 µg + adjuvants	29± 2.6%	47 ± 4.3%	28 ± 4.1%	87 ± 9.1%
GC FA19 vaccine MPs 160 µg + adjuvants	28 ± 2.4%	38 ± 5.1%	31 ± 13.2%	88 ± 12.8%
GC FA19 vaccine MPs 120 µg + adjuvants	25 ± 8.5%	39 ± 2.2%	25 ± 7.5%	80 ± 17.0%
GC FA19 vaccine MPs 80 µg + adjuvants	23 ± 1.7%	47 ± 4.1%	50 ± 0.1%	57 ± 1.2%
GC FA19 vaccine MPs 40 µg + adjuvants	19 ± 1.7%	48 ± 3.3%	60 ± 2%	58 ± 6.0%

## Data Availability

Data are contained within the article and Supplementary Materials.

## Supplementary Materials

The following supporting information can be downloaded at: https://www.mdpi.com/article/10.3390/vaccines12091049/s1. Table S1: Antigen loading in formulating vaccine NPs and MPs against various pathogens, Table S2: Gating strategy for daughter T-cell quantification as they proliferate. Gating strategy and mean FITC-A values for proliferated T-cells in response to nanoparticulated vaccine candidates. The gating values were determined using flow cytometry to quantify T-cell proliferation over a 6-day period. Mean FITC-A values for the proliferated T-cell populations were recorded for the measles vaccine candidate + adjuvants and the *N. gonorrhoeae* vaccine candidate + adjuvants groups at different time intervals. Gating values represent the mean fluorescence intensity corresponding to the different stages of T-cell clonal expansion.
