# Supplementary material for: Evaluating Nanoparticulate Vaccine Formulations for Effective Antigen Presentation and T-Cell Proliferation Using an In Vitro Overlay Assay
Source: Vaccines (Basel). 2024 Sep 13;12(9):1049. doi: 10.3390/vaccines12091049 (PMC11435973; doi:10.3390/vaccines12091049)
Supplement: Supplementary file 1 [file vaccines-12-01049-s001.zip › vaccines-3163687-supplementary.pdf]

Supplementary Materials

Table S1: Antigen loading in formulating vaccine NPs and MPs against various pathogens

Treatment groups	Amount of antigen exposed to the APCs per well	
N. gonorrhoeae CDC F62, FA19 and FA1090 BSA MPs	20 % loading	40 μg – 8 μg per well
Measles, Zika, Influenza A (H1N1), Canine coronavirus PLGA NPs	2 % loading	4 μg- 0.8 μg per well

Table S2: Gating strategy for daughter T-cells quantification as they proliferate

Treatment groups	Days	Gating (Mean FITC-A)
All viral (Measles, H1N1 flu prototype, Canine coronavirus virus and Zika) vaccine candidates + adjuvants	0-1	903845
	1-2	651486
	2-4	498763
	4-6	112981
All *N. gonorrhoeae* (CDC-F62, FA19 and FA1090 strains) vaccine candidates + adjuvants	0-1	954853
	1-2	743856
	2-4	457453
	4-6	269159

Table S2: Gating strategy and mean FITC-A values for proliferated T-cells in response to nanoparticulated vaccine candidates. The gating values were determined using flow cytometry to quantify T-cell proliferation over a 6-day period. Mean FITC-A values for the proliferated T-cell populations were recorded for the measles vaccine candidate + adjuvants and the *N. gonorrhoeae* vaccine candidate + adjuvants groups at different time intervals. Gating values represent the mean fluorescence intensity corresponding to the different stages of T-cell clonal expansion.
